# High efficiency silicon solar cell based on asymmetric nanowire

**DOI:** 10.1038/srep11646

**Published:** 2015-07-08

**Authors:** Myung-Dong Ko, Taiuk Rim, Kihyun Kim, M. Meyyappan, Chang-Ki Baek

**Affiliations:** 1 Department of Electrical Engineering, Pohang University of Science and Technology (POSTECH),77 Cheongam-Ro, Nam-Gu, Pohang, Kyeongbuk, Korea; 2 Department of Creative IT Engineering & Future IT Innovation Lab (POSTECH i-Lab), Pohang University of Science and Technology (POSTECH), 77 Cheongam-Ro, Nam-Gu, Pohang, Kyeongbuk, Korea; 3 NASA Ames Research Center, Moffett Field, CA, 94035

## Abstract

Improving the efficiency of solar cells through novel materials and devices is critical to realize the full potential of solar energy to meet the growing worldwide energy demands. We present here a highly efficient radial p-n junction silicon solar cell using an asymmetric nanowire structure with a shorter bottom core diameter than at the top. A maximum short circuit current density of 27.5 mA/cm^2^ and an efficiency of 7.53% were realized without anti-reflection coating. Changing the silicon nanowire (SiNW) structure from conventional symmetric to asymmetric nature improves the efficiency due to increased short circuit current density. From numerical simulation and measurement of the optical characteristics, the total reflection on the sidewalls is seen to increase the light trapping path and charge carrier generation in the radial junction of the asymmetric SiNW, yielding high external quantum efficiency and short circuit current density. The proposed asymmetric structure has great potential to effectively improve the efficiency of the SiNW solar cells.

Photovoltaic devices using silicon nanowires (SiNW) with a radial p-n junction have received much attention due to their excellent optical and electrical characteristics^1^,^2^,^3^,^4^. Their antireflection properties enhance light absorption, and the orthogonal direction between the charge-carrier collection path and incident light enables the use of low-quality silicon in the production of solar cells^4^,^5^,^6^. The material cost and amenability to eventual large scale fabrication determine the viability of novel concepts in solar cell design in addition to efficiency. In this regard, SiNW radial p-n junction solar cells have been emerging as a promising candidate as they have been greatly improved through various attempts recently. A single p-i-n solar cell was demonstrated first using SiNWs grown by vapor-liquid-solid method aided with gold colloid particles^7^; subsequently, many SiNW arrays using the gold catalytic chemical vapor deposition technique have been reported^8^,^9^. The nanowires can be patterned as needed using different techniques such as electroless etching^10^ and advanced lithography using silica beads^11^,^12^. Light absorption can be enhanced by shaping the structures with antireflection properties such as nanocones^13^,^14^, nanodomes^15^ and nanohemispheres^16^. A passivation layer can be added to improve the surface antireflection properties and enhance the efficiency^17^,^18^. These advances to improve the photovoltaic properties are limited to reducing light reflection at the surface of the solar cell 11,12,13,14,15,16,17,18; however improving the properties using light inside the solar cell has not yet been reported without using a back reflector^4^,^6^.

Here, we present a novel asymmetric SiNW radial p-n junction solar cell to improve the photovoltaic properties using light inside the solar cell by changing the SiNW structure. The optical and electrical properties of the new design are compared directly against those of the symmetric SiNW solar cells. These solar cells were fabricated on a bulk Si wafer using top-down lithography with a dry etching process and poly-silicon as the outermost thin layer of the SiNW. The asymmetric SiNW solar cell shows a maximum short circuit current density (*J*_*SC*_) of 27.5 mA/cm^2^ and an efficiency (*η*) of 7.53%; these figures-of-merit are higher than that for a conventional vertical solar cell (*J*_*SC*_ of 20.4 mA/cm^2^, *η* of 5.26%).

## Results

### Asymmetric SiNW solar cell design

**Figure 1:**
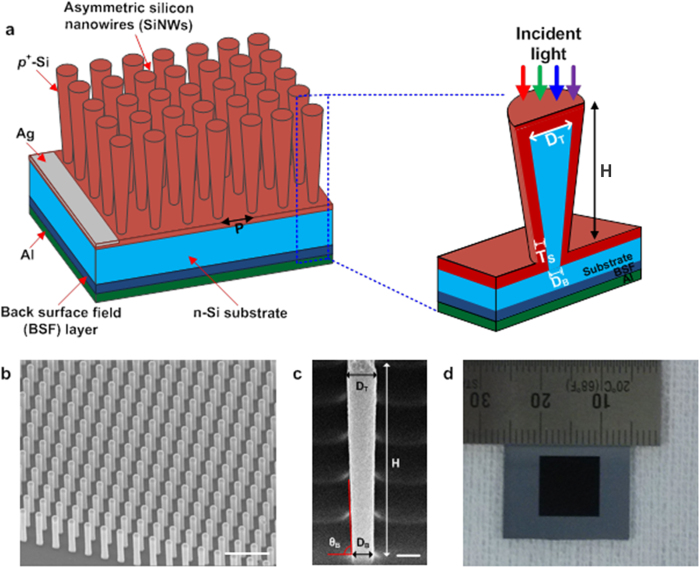
Asymmetric silicon nanowire solar cell. (**a**) Schematic illustration of the asymmetric silicon nanowire (SiNW) solar cell consisting of an array of radial p-n junction asymmetric SiNWs, back surface field (BSF) layer, Al back reflector and Ag top electrode. The asymmetric SiNW has a shorter core diameter (*D*_*B*_) at the bottom than at the top (*D*_*T*_), similar to a funnel or an inverted nanoconical SiNW, while a symmetric SiNW has *D*_*T*_ same as *D*_*B*_. (**b**) Tilted view (45°) SEM image of the SiNW arrays. (**c**) Enlarged view of the asymmetric SiNW with *D*_*T*_ of 370 nm and *D*_*B*_ of 290 nm before p-type shell deposition. A slant bottom angle (*θ*_*B,*_ red) is lower than 90°. (**d**) Optical image of a vertical SiNW solar cell with an area of 1 cm^2^.

Figure 1a shows the schematic illustration of the asymmetric SiNW solar cell. The fabricated device consists of an array of radial p-n junction asymmetric SiNWs, back surface field (BSF) layer, Al back reflector and Ag top electrode. The asymmetric SiNW was designed with its core diameter at the bottom (*D*_*B*_) shorter than at the top (*D*_*T*_), while conventional vertical symmetric SiNW has *D*_*T*_ identical to *D*_*B*_.

The fabrication process of the asymmetric SiNW solar cell consisted of four steps (Supplementary Fig. S1). Starting with 8-inch Si (100) wafers (1–10 Ω · cm, n-type), arsenic ion was implanted with a doping concentration of 10^20^ cm^−3^ in the back side of the Si wafer to form the BSF layer. A thin silicon dioxide (SiO_2_) layer (300 nm) was deposited on the Si wafer as a hard mask layer. A nanodot was patterned on the SiO_2_ layer with an i-line stepper. Then, this nanodot was etched by single step deep reactive ion etching (DRIE) with a mixture of C_4_F_8_ and SF_6_ to form a vertical SiNW. The symmetric and the asymmetric vertical SiNW structures were determined by this etch process (details in the Methods section). A p-type 30 nm silicon with a doping concentration of 10^20^ cm^−3^ was deposited on the SiNW using ultra-high-vacuum chemical vapor deposition to form the p-n junction with uniform thickness over the entire sidewall of a high aspect-ratio SiNW. Finally, A 20/200 nm thick Ti/Ag was deposited on the p-type emitter as the front electrode and a 200 nm thick Al back electrode was deposited to the BSF layer.

The SiNW array shows periodicity with a pitch of 1 *μ*m and a nanowire height (H) fixed at 2.5 *μ*m (Fig. 1b). The asymmetric SiNW arrays were fabricated with three different *D*_*B*_ values of 290, 320, and 350 nm to investigate the impact of asymmetry on the solar cell characteristics. Figure 1c displays the asymmetric SiNW before deposition of the p-type shell. This structure has *D*_T_ = 370 nm, *D*_*B*_ = 290 nm, and *H* = 2.5 *μ*m with *θ*_*B*_ < 90° due to *D*_*B*_ < *D*_*T*_. The radial junction was formed by p-type shells surrounding the cores of SiNWs. Whole cells have the same shell thickness of 30 nm on each sidewall, and the total diameter is increased by twice the shell thickness over the core diameter. Figure 1d shows the fabricated vertical SiNW solar cell with an area of 1 cm^2^ without the front electrode and passivation. We fabricated the solar cells on a 200 mm wafer but the 1-cm^2^ area was chosen for convenience in measurement. The SiNW array (inner square of the solar cell) is darker than the outside of the cell due to low light reflection. Figure S2 under supplementary information provides additional SEM images showing the nanowires and solar cells at various stages.

### Advantages of asymmetric SiNW

**Figure 2:**
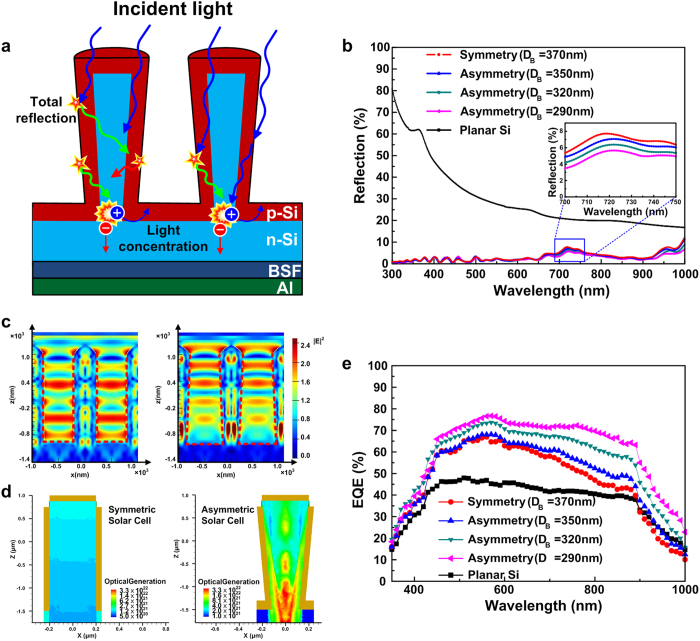
Total reflection effect and optical characteristics of the asymmetric SiNW solar cells. (**a**) Schematic diagram of the total reflection at the sidewall and light concentration in the bottom center of the asymmetric SiNW. (**b**) Light reflection of the planar (black), symmetric SiNW solar cell with *D*_*T*_ of 370 nm (red), and asymmetric SiNW solar cells with *D*_*B*_ of 350 nm (blue) and 320 nm (green). (**c**) Comparison of the field intensity maps (*E*^*2*^) calculated by the FDTD model with a wavelength of 750 nm of the asymmetric SiNW (left, *D*_*T*_ = 370 nm, *D*_*B*_ = 320 nm) and symmetric SiNW (right, *D*_*T*_ = *D*_*B*_ = 370 nm). (**d**) Simulated optical carrier generation rate in the asymmetric and symmetric SiNWs. (**e**) EQE measurements of the symmetric SiNW solar cell with *D*_*T*_ of 370 nm (red), and asymmetric SiNW solar cells with *D*_*B*_ of 350 nm (blue), 320 nm (green), and 290 nm (pink).

The asymmetric structure exhibits different light trapping characteristics compared with the conventional symmetric structure as shown in Fig. 2a. When the incident light proceeds from silicon to air, the refraction angle is much higher than the incident angle due to the difference between the refractive indices of silicon (3.6 ~ 7) and air (1), and light can be totally reflected at the sidewall of the SiNW. Hence, the incident light is continuously reflected in the nanowire until the incident angle becomes lower than a critical angle^19^. Because the angle of re-incidence of the reflected light (green) is lower than the angle of the first incident light (blue), the light trapping path is increased whenever the light is totally reflected at the outer shell of the asymmetric SiNW.

In order to investigate the light trapping properties of the asymmetric SiNW solar cells, we measured four types of solar cells: symmetric SiNW with diameter of 370 nm, and three asymmetric SiNWs with the same *D*_*T*_ of 370 nm but different *D*_*B*_ values of 350 nm, 320 nm, and 290 nm. Figure 2b shows the light reflection of all types of solar cells as a function of wavelength ranging from 300 to 1000 nm. As the shape of the SiNW changes from symmetric to asymmetric (decreasing *D*_*B*_), the light reflection is totally decreased due to the increase of light trapping path. Because the top surfaces of all SiNWs mainly related to the total light reflection are identical, the asymmetric SiNW solar cell shows a slight decrease in light reflection compared to the symmetric SiNW solar cell. Figure 2b also shows that the all SiNW structures have lower reflection than the planar silicon surface over the entire wavelength range, which is consistent with previous theoretical and experimental results^5^,^6^.

In addition, the reflected light proceeds towards the bottom center of the asymmetric SiNW, and the angle between the re-incident light and horizontal plane decreases whenever it reflects, like as if a green wave is changed to a red wave in Fig 2a. These phenomena enhance the proportion of incident light passing through the radial junction, which causes the light concentration in the bottom center of the asymmetric SiNW (also in Fig. 2a). Figure 2c shows the simulated electric field intensity distributions of solar cells with symmetric SiNW (right pictures, *D*_*T*_ = *D*_*B*_ = 370 nm) and asymmetric SiNW (left pictures, *D*_*T*_ = 370 nm, *D*_*B*_ = 320 nm) under the incident light with a wavelength of 750 nm. The electric field intensity was calculated using a three-dimensional finite-difference time-domain (FDTD) simulator (Lumerical Solution, Inc.). The simulation results show that the asymmetric SiNW has higher electric field intensity than the symmetric SiNW due to the light concentration. The bottom center of the asymmetric SiNW displays a red color (*E*^*2*^ > 2.5) in contrast to green for the symmetric SiNW. Consequently, the high electric field intensity causes an increase in the charge carrier generation rate as shown in Fig. 2d, which can be verified thorough external quantum efficiency (EQE) of the solar cells.

Figure 2e shows the EQE of the symmetric SiNW and three asymmetric SiNW solar cells. The EQE was measured in the visible and near IR regime using a monochromater and semiconductor parameter analyzer. Going from symmetric to asymmetric, the EQE increases over the entire wavelength regime. In addition, the relative ratio between the EQEs grows as the wavelength increases. The asymmetric SiNW solar cell with minimum *D*_*B*_ (*D*_*B*_ = 290 nm, pink triangle) exhibits an EQE of 72 %, whereas it is 52 % for the symmetric SiNW solar cell (red circle) at *λ* = 750 nm. On the other hand, at *λ* = 488 nm, the EQE of the asymmetric SiNW with minimum *D*_*B*_ and symmetric SiNW reaches 69% and 60%, respectively. As the wavelength increases, the light transmitted to the bottom radial junction of the SiNW increases, which causes the improvement in the EQE of the asymmetric SiNW solar cell.

### Performance of asymmetric SiNW design in Si solar cells

**Figure 3:**
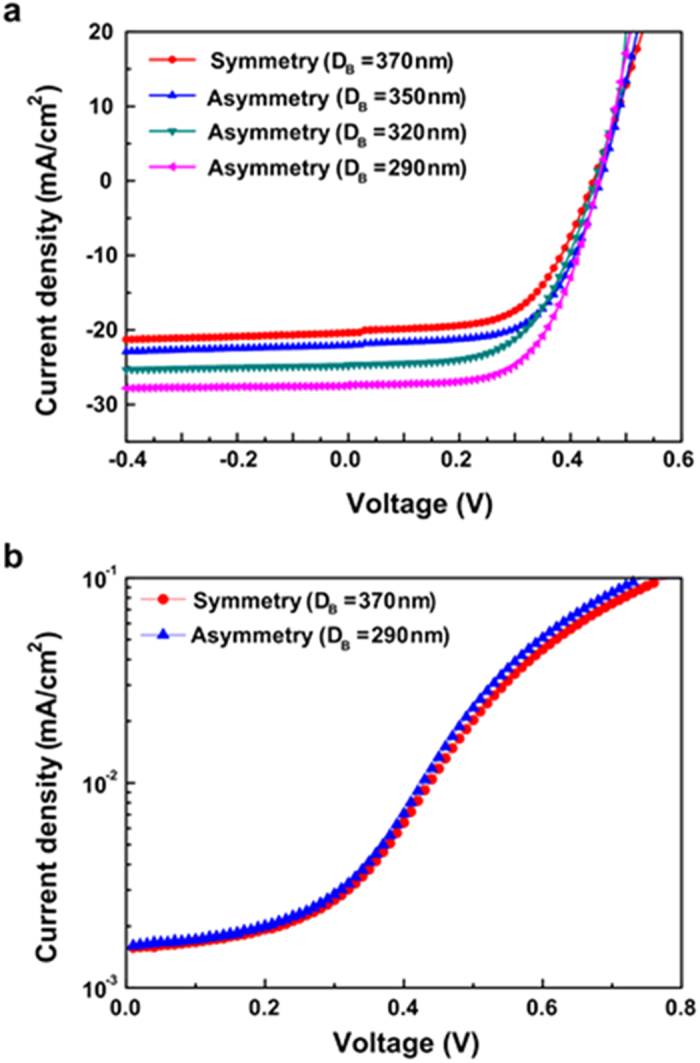
Performance of the asymmetric SiNW solar cells. (**a**) Measured output current density-voltage curves under A.M1.5G illumination of the symmetric SiNW solar cell with *D*_*T*_ of 370 nm (red), and asymmetric SiNW solar cells with *D*_*B*_ of 350 nm (blue), 320 nm (green), and 290 nm (pink). (**b**) Current density-voltage curves under dark condition of the symmetric SiNW solar cell (red), and asymmetric SiNW solar cells with *D*_*B*_ of 290 nm (blue).

The *J-V* characteristics are presented in Fig. 3a and Table 1 for four types of SiNW solar cells under AM 1.5 G illumination with 100 mW/cm^2^, which show that the electrical properties of the SiNW solar cell are improved by shrinking the *D*_*B*_ of the SiNW. When *D*_*B*_ of the SiNW cells decreases from 370 to 350, 320, and 290 nm, their short circuit current density (*J*_*SC*_) increases from 20.4 to 22.1, 24.8, and 27.5 mA/cm^2^, respectively, and the efficiency (*η*) also increases from 5.26 to 6.11, 6.37, and 7.53%, respectively.

**Figure 4:**
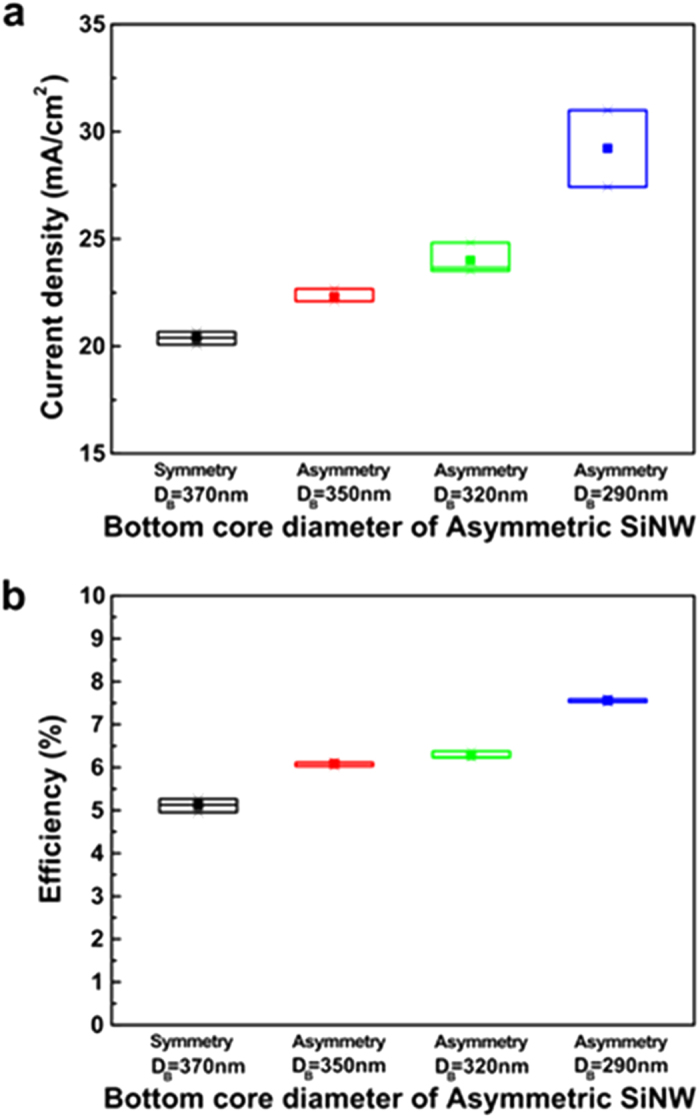
Photovoltaic properties of symmetric, and asymmetric SiNW solar cells. (**a**) The distribution of the measured short circuit current density of the symmetric SiNW (green) and asymmetric SiNW solar cells. (**b**) The distribution of the efficiency of four types of SiNW solar cells. Each distribution comes from the J-V characteristics of the solar cells fabricated with identical design and process parameters.

The asymmetric SiNW cell with minimum *D*_*B*_ (*D*_*B*_ = 290 nm) has 1.3 times higher *J*_*SC*_ and *η* than those of the symmetric SiNW solar cell. Due to similar *V*_*OC*_ (0.44 ~ 0.455) and FF (0.59 ~ 0.62) for the four types of SiNW solar cells, the enhanced *J*_*SC*_ mainly contributes to the increase in the *η* of those cells. In other words, the dark current of the symmetric and the asymmetric SiNW solar cells are very similar (see Fig. 3b). Therefore, the improvement in *J*_*SC*_ comes from the increase in the EQE due to the enhanced charge carrier generation in the radial junction as *D*_*B*_ decreases. Figure 4 shows the distribution of the *J*_*SC*_ and *η* of the solar cells, indicating that the *J*_*SC*_ and *η* of the SiNW cells can be improved by shrinking the *D*_*B*_ of the SiNW even if there are differences between the characteristics of the cells with same structure.

### Discussion

We have demonstrated an asymmetric SiNW solar cell with enhanced photovoltaic properties through shaping the nanowires with a shorter core diameter at the bottom compared to the top. This cell exhibits higher *J*_*SC*_ than the symmetric SiNW solar cells stemming from the increase in the light trapping path and the charge carrier generation in the radial junction due to total reflection at the sidewall of the asymmetric SiNW. Though the focus here is to demonstrate the superiority of the proposed configuration in direct comparison with symmetric NW cells, the absolute efficiency can be improved through multiple routes. For example, we patterned the metallic top contacts to reduce the contact resistance on the SiNW array. All of the electrical results show a high series resistance causing a low fill factor (~0.6). The front contact pattern consists of several finger bars and bus bars; the series resistance is considerably affected by the specific design of these bars including the width, thickness, the gap between the bars and the number of bars. We can improve the absolute efficiency of the cells by increasing the fill factor via optimization of the front pattern design.

The open-circuit voltage (VOC) depends on the wafer doping concentration and thickness. We fabricated the SiNW solar cell here on a bulk integrated circuit (IC) grade-Si wafer with 700-nm thickness and a resistivity of 1 ~ 10 Ω · cm to show that our solar cells are fabricated using conventional IC fabrication technologies. The minimum minority carrier diffusion length is determined by the wafer thickness because these cells use a back contact. Finally, an 80 nm antireflection layer on the front SiNW array even in the asymmetric pattern can provide additional increase in current density. The novel structure demonstrated here is expected to offer an avenue to enhance the efficiency of SiNW solar cells.

## Methods

### Si solar cell fabrication process

An n-type 700-μm thick 200 mm Czochralski grade silicon (100) wafer with a resistivity of 1 ~ 10 Ω · cm was used after a cleaning process. Highly doped arsenic ions (1 × 10^20^ cm^−3^) were diffused into the backside of the target substrates to reduce the carrier recombination at the interface between the back metal contact and n-type silicon substrate and also the series resistance. Then the wafers were annealed by rapid thermal annealing at 1000 °C for 2 hours to remove any damage from the ion implantation and to activate the dopants. This annealing process was conducted before nanowire formation to protect the dopant activation of the n-type shell during the backside annealing process. A 7-inch reticle composed of several 1 cm × 1 cm cells and i-line stepper were used to define the pattern of the silicon nanowire (SiNW). After exposure, the wafers were baked at 110 °C for 1 min, and developed in AZ 300 MIF for 1 min. The patterned wafers were etched using DRIE with single step DRIE process (SF_6_/C_4_F_8_/Ar) in order to make vertically aligned symmetric and asymmetric SiNWs. The 30-nm p-type shell with doping concentration of 10^20^ cm^−3^ was formed by a mixture of SiH_4_ and B_2_H_6_ at 700 °C for 5 min. The front contact was made by Ti/Ag deposition and lift-off process. The front contact pattern consists of finger bars and bus bars in order to reduce the series resistance. The bar pattern was defined by mask aligner with 365 nm UV exposure and developed in AZ 300 MIF for 1 min. After patterning of the photoresist layer, the 20-nm Ti and 200-nm Ag layers were deposited on the front surface using electron beam evaporator. Then, a 3-μm thick photoresist was deposited on the front surface to protect the SiNW and the metal pattern from the damage for backside metal deposition process. The wafers were shortly soaked in diluted HF solution to remove SiO_2_ of the backside before a 200-nm thick Al was deposited on the backside of Si wafers using electron beam evaporator. Finally, the wafers were annealed at 400 °C for 10 min to reduce the recombination at the back contact and obtain ohmic contact.

### Asymmetric SiNW fabrication process

The shape of the SiNW and a slant bottom angle (*θ*_*B*_) between the substrate and sidewall were controlled by the flow rates of SF_6_ and C_4_F_8_. Because SF_6_ and C_4_F_8_ perform the roles of selective etching and sidewall passivation respectively, the etching process dominated by SF_6_ led to the top diameter being lower than the bottom diameter (*θ*_*B*_ > 90) such as in a nanocone. In contrast, the etching process dominated by C_4_F_8_ resulted in larger diameter at the top compared to the bottom (*θ*_*B*_ < 90), similar to an inverted nanocone or a funnel. A gas mixture of SF_6_/C_4_F_8_/Ar was used with a rate of 35/65/40 sccm to make the baseline vertical symmetric SiNWs with *D*_*T*_ = 370 nm. As the flow rate of SF_6_/C_4_F_8_ is changed to 37/63, 39/61, and 40/60 sccm, the asymmetric SiNWs with *D*_*B*_ of 350 nm, 320 nm, and 290 nm were fabricated, respectively (keeping *D*_*T*_ = 370 nm). Consequently, the symmetric and asymmetric SiNW could be achieved by simply controlling the flow rates of etch gases.

### Optical measurements

The light reflection was measured with an UV/Vis spectrophotometer (Cary 5000, Varian). Equivalent apertures on both the sample and blank were used to ensure the reliability of the measurements. The baseline (blank sample) was measured before the sample measurement and used for the correction of the sample measurements. External quantum efficiency (EQE) measurements were made with a monochromater (Cornerstone 130, Newport) and a xenon lamp (150 W, Newport). The output current signal was measured using an Aglient 4156C semiconductor parameter analyzer. For all EQE measurements, the values were calculated by using different current densities between the illumination and dark conditions, and the measurement condition of the monochromater was controlled by a Lab-view software.

### Optical Simulation

A three-dimensional finite-difference time-domain (FDTD) simulation (Lumerical Solution, Inc.) was used to calculate the field intensity of the symmetric and asymmetric SiNWs. The simulated structures were identical to the actual fabricated cells. We simulated an area of 6 μm by 6 μm with 5 μm thickness (25 SiNWs, 1 μm period between SiNWs. Supplementary Fig. S3) due to limitations of computational time. The material properties of silicon were taken from the database of Palik in the FDTD simulator. The light source was placed 500 nm above the SiNWs and in the center of the simulated region, which in turn can illuminate the whole area of the simulated device.

### Electrical measurements

The electrical characteristics of the solar cells were measured with a solar simulator (450 W Class AAA solar simulator, Newport) and a dual-channel system source meter instrument (Keithley 2636A, Keithley). The solar simulator consisted of a 450 ARC lamp, exposure control instrument and a reference cell. The light with an intensity up to 100 mW/cm^2^ illuminated the cells through the AM1.5G filter. The reference cell was used to ensure the intensity of the simulator. Before measuring the dark and illumination current-voltage curves, the probe tips were connected to the SMUs of the source meter. The input voltage signal of the probe tip was controlled by the source meter with Lab-view software.

## Additional Information

**How to cite this article**: Ko, M.-D. *et al.* High efficiency silicon solar cell based on asymmetric nanowire. *Sci. Rep.* **5**, 11646; doi: 10.1038/srep11646 (2015).

## Supplementary Material

Supplementary Information

## Figures and Tables

**Table 1:** Summary of the average *J-V* characteristics of four types of SiNW solar cells.

Structure	*J*_*SC*_ (mA/cm^2^)	*V*_*OC*_ (V)	Fill Factor	Efficiency (%)
Symmetric SiNW (*D*_*B*_ = 370 nm)	20.4	0.44	0.59	5.26
Asymmetric SiNW (*D*_*B*_ = 350 nm)	22.1	0.45	0.62	6.11
Asymmetric SiNW (*D*_*B*_ = 320 nm)	24.8	0.445	0.58	6.37
Asymmetric SiNW (*D*_*B*_ = 290 nm)	27.5	0.455	0.60	7.53
