# Supplementary material for: High efficiency silicon solar cell based on asymmetric nanowire
Source: Sci Rep. 2015 Jul 8;5:11646. doi: 10.1038/srep11646 (PMC4495391; doi:10.1038/srep11646)
Supplement: Supplementary Information [file srep11646-s1.doc]

**Supplementary Information**

**High efficiency silicon solar cell based on asymmetric nanowire**

Myung-Dong Ko^1^, Taiuk Rim^2^, Kihyun Kim^2^, M. Meyyappan^2,3^, & Chang-Ki Baek^1,2,*^,

^1^Department of Electrical Engineering and ^2^Department of Creative IT Engineering, Pohang University of Science and Technology (POSTECH), 77 Cheongam-Ro, Nam-Gu, Pohang, Kyeongbuk, Korea

^3^NASA Ames Research Center, Mountain View, CA, 94035

*E-mail :baekck@postech.ac.kr

**Supplementary FigureS1.** Fabrication process of the symmetric and asymmetric SiNW solar cells. First, backside of the n-type Si wafer was ion implanted to form back-surface-field layer and silicon oxide was deposited on the substrate as a hardmask. A photoresist was deposited on the oxide layer and patterned by photolithography. Then, the silicon oxide was etched by a selective etching process and photoresist with a nanodot shape. The symmetric and asymmetric SiNWs were formed by deep reactive ion etching with the silicon oxide hard mask layer using C_4_F_8_ and SF_6_ gas flow. Then, p-type poly-silicon was deposited to uniformly form the p-n junction layer. Finally, Ti/Ag was metallized as a front contact pad and Al was deposited on the backside of the n-Si wafer to make the backside contact pad.


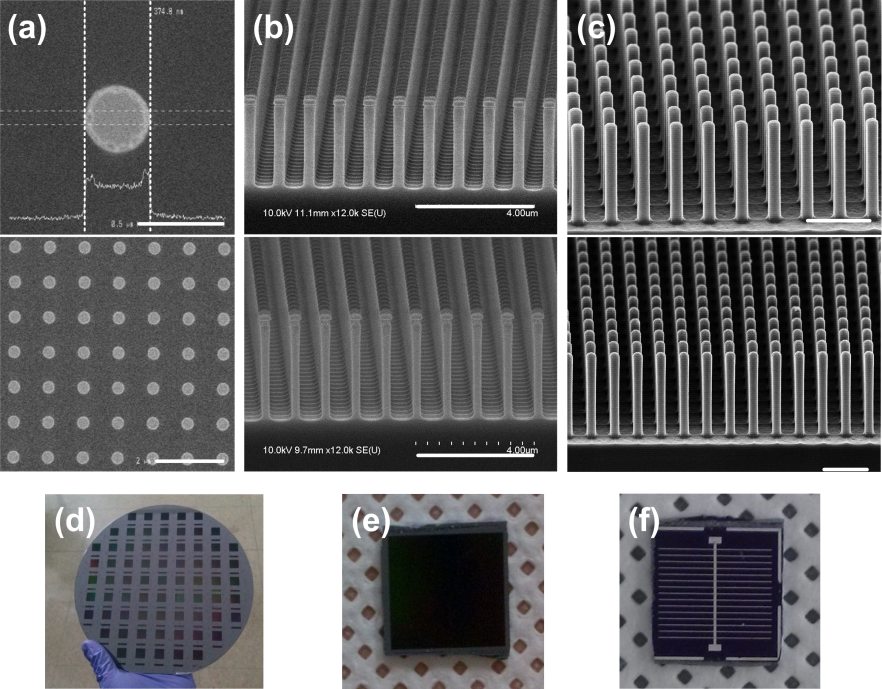


**Supplementary FigureS2.** (a) Top-view SEM images of the oxide nanodot pattern made by top-down lithography. (b) Cross-sectional SEM images of the symmetric and asymmetric SiNW after selective etching. (c) Cross-sectional SEM images of the symmetric and asymmetric SiNW after p-Si deposition using UHV-CVD. (d) Optical image of the 8-inch wafer with the vertical SiNW pattern, (e) fabricated SiNW solar cell before contact formation, and (f) fabricated vertical SiNW solar cell with an area of 1cm^2^.

**Supplementary FigureS3.** (a) Schematic illustration of the FDTD simulated structure, consisting of 25 silicon nanowires and the substrate. (b) Enlarged view of the simulated structure. Enlarged views of a single (c) symmetric SiNW and (d) asymmetric SiNW.
